# Nutrition literacy differs based on demographics among University students in Bengbu, China

**DOI:** 10.3389/fpubh.2023.1113211

**Published:** 2023-03-03

**Authors:** Tianjing Gao, Ying Duan, Qi Qi, Guangju Mo, Siyue Han, Huaqing Liu, Min Zhang

**Affiliations:** ^1^School of Public Health, Bengbu Medical College, Bengbu, Anhui, China; ^2^School of Health Management, Bengbu Medical College, Bengbu, Anhui, China

**Keywords:** nutrition literacy, nutrition assessment, healthy diet, university student, China

## Abstract

**Background:**

Nutrition literacy (NL) encompasses the knowledge and skills that inform individuals' food choices. This cross-sectional study explored factors associated with NL among Chinese university students in Bengbu, China.

**Methods:**

A cross-sectional survey was carried out. Two thousand one hundred thirty-three university students were selected by stratified cluster sampling. A 43-item NL questionnaire was used to assess NL. Binary logistic regression was used to determine odds ratios (ORs) along with 95% confidence intervals (CIs) for NL and to test the interaction effects of multiple factors on total NL and its six dimensions.

**Results:**

Of these participants, 1,399 (65.6%) were women and 734 (34.4%) were men. Students who were from urban areas (OR = 1.36, 95% CI: 1.08–1.72), were living with both parents (OR = 1.30, 95% CI: 1.02–1.65), and had high academic performance (OR = 1.85, 95% CI: 1.34–2.57) were more likely to report higher NL levels than did other students. The ORs for NL (OR = 1.60, 95% CI: 1.06–2.41), nutrition knowledge (OR = 1.51, 95% CI: 1.00–2.26), obtaining skills (OR = 1.76, 95% CI: 1.16–2.65), and critical skills (OR = 1.59, 95% CI: 1.05–2.39) were higher for medical students who had received nutrition education than for other students. The ORs for NL (OR = 2.42, 95% CI: 1.21–4.84), nutrition understanding (OR = 2.59, 95% CI: 1.28–5.25), and interactive skills (OR = 2.06, 95% CI: 1.04–4.08) were higher for only-child students and those with a monthly expenditure of >¥1500.

**Conclusions:**

NL of university students differed in terms of place of origin, living arrangement, nutrition education, academic performance, and household income, and the findings imply that universities should have all students take a basic nutrition course to improve their NL.

## 1. Introduction

The period of university study may be influential in the establishment of long-term dietary patterns and may thus influence the risk of chronic diseases (1). The transition from high school to university, known as emergent adulthood, is a vulnerable period that is frequently characterized by weight gain; therefore, it is a critical period for prevention and intervention in relation to dietary patterns (2, 3). This period of transition is typically characterized by leaving home for the first time, adapting to a new environment, developing new friendships and social networks, and having greater independence in overall decision-making (4, 5). An individual's dietary attitudes and behaviors during the period of university study can profoundly influence their adult lifestyle habits and thus influence the risk of obesity and related comorbidities such as diabetes and heart disease (6). University students constitute a vulnerable group for poor dietary intake, insufficient physical activity, and sedentary behavior (7). Young people are usually prone to adopt unhealthy dietary habits (8). They exhibit dietary restraint, low intake of fruit and vegetables, and high intake of energy-dense nutrient-poor foods such as takeaway foods and sugar-sweetened drinks (5, 9). Factors influencing healthy eating among university students include individual factors (e.g., nutrition knowledge and education), social factors (e.g., social support from parents), and environmental factors (e.g., product prices and limited budgets) (10, 11).

To reduce the increasing prevalence of nutritional health problems, it is of great importance to increase the knowledge level of individuals and society about nutrition and to develop healthy nutrition skills and behaviors (12). Nutrition literacy (NL), also known as “health literacy applied in the field of nutrition” (13), refers to individuals' competence in healthy eating. NL was defined as “the degree to which individuals have the capacity to obtain, process, and understand nutrition information and skills needed in order to make appropriate nutrition decisions” (14). Studies present nutrition literacy measurement instruments should be of multiple characteristics, e.g., different domains of cognition and skill, different dimensions of obtain, understand, analyze, appraise, and apply (15–17). However, existing NL instruments often assessed a certain characteristic of NL; moreover, they mainly focused on functional nutrition literacy (13) and rarely included interactive or critical nutrition literacy. Our previous study (18) developed a nutrition literacy measurement instrument with multiple characteristics which assess comprehensively NL for Chinese adults. Our NL instrument include two domains (cognition and skill), 3 levels of nutrition literacy (functional, interactive, and critical) and 6 dimensions of knowledge, understanding, obtaining skills, applying skills, interactive skills (the ability to act effectively to improve health and to communicate, provide, and apply relevant health information), and critical skills (the ability to critically assess and reflect on nutritional information or dietary advice in terms of personal nutritional needs) (13, 15). The dimensions of nutrition knowledge and nutrition understanding represent the understanding of nutrition information and services; the dimension of obtaining skills represents the process of obtaining nutrition information and services; and the dimensions of applying skills, interactive skills, and critical skills represent the processing and application of nutrition information and services (18, 19). Therefore, NL encompasses the crucial knowledge and skills that inform food choices (20). NL emphasizes nutrition-related skills in which an individual should have to make wise decisions regarding dietary situations in daily life, it can be regarded as an imperative component of food education programs and important to promoting healthy eating behaviors (21). A university campus with an adequate eating environment and adequate healthy eating campaigns could effectively improve healthy eating behaviors in university students (11). Higher levels of NL were reported to be associated with healthier and higher-quality diets, which could in turn reduce the risk of diet-related chronic diseases (22).

A handful of studies have been conducted on NL among different subgroups of the population (i.e., adolescents, students, and adults) in Turkey (23, 24), Taiwan (25), Iran (26), US (27, 28), and Palestine (29). Nevertheless, there is a lack of available evidence to investigate the NL of Chinese university students. Accordingly, the presented study investigated factors associated with NL and its six dimensions among university students in China. The findings of this study may inform the design of interventions for improving NL among university students.

## 2. Materials and methods

### 2.1. Participants and procedure

This study involved a cross-sectional design and was conducted from April to June 2021 in Bengbu, China. Participants were recruited through stratified cluster sampling. Firstly, two universities (medical and non-medical) were selected by convenience sampling. Second, eight classes were randomly selected in each grade. And then all students (about 30 individuals) in these classes were asked to participate in the survey. An individual who was 18 years old and above was included in the survey if he or she willing to participate in it, but was excluded if he or she was unwilling to do it. The students were notified that participation was voluntary, and signed informed consent was obtained. This study was approved by the Ethics Committee of Bengbu Medical College. A total of 2,190 students completed the survey. After the exclusion of 57 (2.7%) students who provided invalid responses, 2,133 students remained, and their responses were analyzed in this study.

### 2.2. NL assessment

A 43-item NL questionnaire (NL-43) (18), which was developed by experts in public health and nutrition education and promotion using the Delphi method, was used to assess the students' NL in the six dimensions containing nutrition knowledge (7 items), nutrition understanding (5 items), obtaining skills (5 items), applying skills (11 items), interactive skills (9 items), and critical skills (6 items). The Cronbach's alpha for the total scale of NL was 0.962 and the Cronbach's alpha for each dimensional scale ranged from 0.845 to 0.954. Each item is rated on a 5-point Likert-type scale (1 = strongly disagree, 2 = disagree, 3 = average, 4 = agree, 5 = strongly agree; or 1 = strongly non-conforming, 2 = non-conforming, 3 = average, 4 = conform, 5 = strongly conform), with a higher score indicating a higher NL level. The total score for the NL Scale is 215. NL and its six dimensions were dichotomised into low and high levels on the basis of their corresponding median scores (Supplementary Table 1). An individual was referred as high levels (coded as 1) in NL and its six dimensions if the score was above corresponding median score; otherwise, was referred as low levels (coded as 0).

### 2.3. Demographic information

This study obtained the students' demographic information, including their age (classified as 16–21 vs. 22–27 years), sex (male vs. female), major (medical vs. non-medical majors), grade (junior vs. senior), place of origin (rural vs. urban), only-child status (yes vs. no), living arrangement (living with both parents vs. living with a single parent, grandparents, or other). Acquisition of nutrition education was obtained through the question “Did you take any courses in nutrition at school?”; responses were “no” (coded as 1), “yes” (coded as 2). Parent education level was classified as elementary school or below (coded as 1), junior high school (coded as 2), high school or technical school (coded as 3), and college or university and above (coded as 4). Academic performance was obtained using the question “What was your grade point average in the last semester?”; responses were “ <70” as “poor,” “70–80” as “average,” and “≥80” as “good.” Household income per month was classified as <¥6,000 (coded as 1), ¥6,000–12,000 (coded as 2), and >¥12,000 (coded as 3). Expenditure per month was classified as <¥1,000 (coded as 1), ¥1,000–1,500 (coded as 2), and >¥1,500 (coded as 3).

### 2.4. Statistical analysis

All data were entered in duplicate into an Epi Data version 3.1 database (EpiData Association, Odense Denmark). Measurement data are presented herein as mean (M) ± standard deviation (SD). Descriptive statistics were used to determine the distributions of total NL and its six dimensions. Moreover, categorical variables are expressed herein as numbers and proportions, and such variables were compared across groups by using a chi-square test. Ddependant variable (NL) is dichotomous, and binary logistic regression was performed to determine the odds ratios (ORs) and the corresponding 95% confidence intervals (CIs) for NL; it was also conducted to evaluate the interaction effects of multiple factors on total NL and its six dimensions. Some independent variables had three codes or above and were put into model as categorical. All statistical analyses were performed using SPSS 26.0 (IBM, Armonk, NY, USA). *P* < 0.05 (two-sided) was considered statistically significant.

## 3. Results

As presented in Table 1, this study included a total of 2,133 university students, and their age ranged from 16 to 27 years (M = 20.91; SD = 1.57). Of these students, 65.6% were women and 34.4% were men. Furthermore, 47.0% of the participants were medical students, and 53.0% were nonmedical students. Of the students, 49.9% were seniors, 69.6% were from rural areas, 29.2% were the only child in the family, 83.8% were living with both parents, 53.4% had received nutrition education, 42.7% had high academic performance, 9.8% belonged to households with a monthly income of >¥12,000, and 25.1% had a monthly expenditure of >¥1,500. Most of the students reported that their parents' education level was junior high school (with 46.7% of fathers and 40.6% of mothers attaining this education level).

**Table 1:** Distribution of different characteristics by total nutrition literacy and its six dimensions.

**Characteristics**	***n* (%)**	**Nutrition literacy**	***χ^2^***	**Knowledge**	***χ^2^***	**Understanding**	***χ^2^***	**Obtaining skills**	***χ^2^***	**Applying skills**	***χ^2^***	**Interactive skills**	***χ^2^***	**Critical skills**	***χ^2^***
Age group (years)			3.955 ^*^		0.384		14.891 ^***^		4.538 ^*^		3.945 ^*^		2.945		9.530 ^**^
16–21	1,428 (66.9)	676 (47.3)		705 (49.4)		597 (41.8)		597 (41.8)		668 (46.8)		689 (48.2)		624 (43.7)	
22–27	705 (33.1)	366 (51.9)		338 (47.9)		357 (50.6)		329 (46.7)		362 (51.3)		368 (52.2)		358 (50.8)	
Sex			0.002		1.609		4.725 ^*^		0.242		0.741		0.327		12.768 ^***^
Male	734 (34.4)	359 (48.9)		345 (47.0)		352 (48.0)		324 (44.1)		345 (47.0)		370 (50.4)		377 (51.4)	
Female	1,399 (65.6)	683 (48.8)		698 (49.9)		602 (43.0)		602 (43.0)		685 (49.0)		687 (49.1)		605 (43.2)	
Major			14.595 ^***^		27.613 ^***^		67.840 ^***^		8.632 ^**^		0.021		9.205 ^**^		17.625 ^***^
Medical	1,003 (47.0)	534 (53.2)		551 (54.9)		543 (54.1)		469 (46.8)		486 (48.5)		532 (53.0)		510 (50.8)	
Non-medical	1,130 (53.0)	508 (45.0)		492 (43.5)		411 (36.4)		457 (40.4)		544 (48.1)		525 (46.5)		472 (41.8)	
Grade			5.160 ^*^		0.056		15.442 ^***^		3.080		0.637		1.416		13.412 ^***^
Junior	1,069 (50.1)	496 (46.4)		520 (48.6)		433 (40.5)		444 (41.5)		507 (47.4)		516 (48.3)		450 (42.1)	
Senior	1,064 (49.9)	546 (51.3)		523 (49.2)		521 (49.0)		482 (45.3)		523 (49.2)		541 (50.8)		532 (50.0)	
Place of origin			28.751 ^***^		11.264 ^**^		29.856 ^***^		14.605 ^***^		16.100 ^***^		18.253 ^***^		26.198 ^***^
Rural	1,484 (69.6)	668(45.0)		690(46.5)		606(40.8)		604(40.7)		674(45.4)		690(46.5)		629(42.4)	
Urban	649 (30.4)	374 (57.6)		353 (54.4)		348 (53.6)		322 (49.6)		356 (54.9)		367 (56.5)		353 (54.4)	
Only-child status			12.867 ^***^		0.635		12.124 ^***^		10.381 ^**^		9.392 ^**^		15.452 ^***^		18.630 ^***^
No	1,510 (70.8)	700 (46.4)		730 (48.3)		639 (42.3)		622 (41.2)		697 (46.2)		707 (46.8)		650 (43.0)	
Yes	623 (29.2)	342 (54.9)		313 (50.2)		315 (50.6)		304 (48.8)		333 (53.5)		350 (56.2)		332 (53.3)	
Living arrangement			9.746 ^**^		8.442 ^**^		2.117		1.634		8.377 ^**^		8.620 ^**^		1.086
Living with both parents	1,788 (83.8)	900 (50.3)		899 (50.3)		812 (45.4)		787 (44.0)		888 (49.7)		911 (51.0)		832 (46.5)	
Living with a single parent, grandparents, or other	345 (16.2)	142 (41.2)		144 (41.7)		142 (41.2)		139 (40.3)		142 (41.2)		146 (42.3)		150 (43.5)	
Father's education level			16.011 ^**^		2.669		31.381 ^***^		13.135 ^**^		12.835 ^**^		11.585 ^**^		21.812 ^***^
Elementary school or below	371 (17.4)	160 (43.1)		191 (51.5)		143 (38.5)		139 (37.5)		163 (43.9)		162 (43.7)		157 (42.3)	
Junior high school	996 (46.7)	468 (47.0)		469 (47.1)		419 (42.1)		425 (42.7)		459 (46.1)		484 (48.6)		423 (42.5)	
High school or technical school	452 (21.2)	236 (52.2)		226 (50.0)		210 (46.5)		202 (44.7)		236 (52.2)		236 (52.2)		228 (50.4)	
College or university and above	314 (14.7)	178 (56.7)		157 (50.0)		182 (58.0)		160 (51.0)		172 (54.8)		175 (55.7)		174 (55.4)	
Mother's education leve>p27mm			22.738 ^***^		7.633		28.920 ^***^		14.897 ^**^		13.817 ^**^		14.853 ^**^		23.617 ^***^
Elementary school or below	727 (34.1)	324 (44.6)		346 (47.6)		283 (38.9)		287 (39.5)		327 (45.0)		332 (45.7)		305 (42.0)	
Junior high school	866 (40.6)	408 (47.1)		410 (47.3)		384 (44.3)		375 (43.3)		407 (47.0)		421 (48.6)		386 (44.6)	
High school or technical school	345 (16.2)	194 (56.2)		192 (55.7)		173 (50.1)		158 (45.8)		184 (53.3)		191 (55.4)		174 (50.4)	
College or university and above	195 (9.1)	116 (59.5)		95 (48.7)		114 (58.5)		106 (54.4)		112 (57.4)		113 (57.9)		117 (60.0)	
Acquisition of nutrition education			27.205 ^***^		14.290 ^***^		40.630 ^***^		5.168 ^*^		9.493 ^**^		13.336 ^***^		30.174 ^***^
No	995 (46.6)	426 (42.8)		443 (44.5)		372 (37.4)		406 (40.8)		445 (44.7)		451 (45.3)		395 (39.7)	
Yes	1,138 (53.4)	616 (54.1)		600 (52.7)		582 (51.1)		520 (45.7)		585 (51.4)		606 (53.3)		587 (51.6)	
Academic performance			28.284 ^***^		4.865		14.844 ^**^		18.055 ^***^		25.425 ^***^		19.722 ^***^		10.060 ^**^
Poor	193 (9.0)	78 (40.4)		92 (47.7)		75 (38.9)		74 (38.3)		73 (37.8)		79 (40.9)		82 (42.5)	
Average	1,030 (48.3)	460 (44.7)		481 (46.7)		429 (41.7)		409 (39.7)		464 (45.0)		479 (46.5)		445 (43.2)	
Good	910 (42.7)	504 (55.4)		470 (51.6)		450 (49.5)		443 (48.7)		493 (54.2)		499 (54.8)		455 (50.0)	
Household income per month (RMB)			11.989 ^**^		3.515		21.291 ^***^		11.701 ^**^		9.478 ^**^		3.180		5.373
< 6,000	1,043 (48.9)	487 (46.7)		501 (48.0)		433 (41.5)		439 (42.1)		490 (47.0)		501 (48.0)		463 (44.4)	
6,000−12,000	881 (41.3)	430 (48.8)		427 (48.5)		398 (45.2)		373 (42.3)		418 (47.4)		442 (50.2)		408 (46.3)	
>12,000	209 (9.8)	125 (59.8)		115 (55.0)		123 (58.9)		114 (54.5)		122 (58.4)		114 (54.5)		111 (53.1)	
Expenditure per month (RMB)			3.854		2.962		15.178 ^**^		10.714 ^**^		1.506		1.924		4.368
< 1,000	275 (12.9)	131 (47.6)		126 (45.8)		118 (42.9)		122(44.4)		141 (51.3)		134 (48.7)		125 (45.5)	
1,000−1,500	1,323 (62.0)	630 (47.6)		640 (48.4)		558 (42.2)		541 (40.9)		627 (47.4)		644 (48.7)		590 (44.6)	
>1,500	535 (25.1)	281 (52.5)		277 (51.8)		278 (52.0)		263 (49.2)		262 (49.0)		279 (52.1)		267 (49.9)	

* *P* < 0.05,

** *P* < 0.01,

*** *P* < 0.001.

The univariate analysis results revealed significant differences in total NL by age, major, grade, place of origin, only-child status, living arrangement, acquisition of nutrition education, academic performance, and household income per month (Table 1). Among the participants, medical students exhibited significantly higher total NL than did non-medical students. The results also indicated a significant relationship between total NL levels and parents' education level; specifically, students whose parents' education level was college or university and above exhibited the highest total NL level. Additionally, the levels of critical skills were significantly associated with sex. Specifically, male students had higher levels of critical skills than did female participants (51.4 and 43.2%, respectively).

Multiple logistic regression was performed to determine factors influencing total NL and its six dimensions (Table 2). The results revealed that students who were from urban areas (OR = 1.36, 95% CI: 1.08–1.72), were living with both parents (OR = 1.30, 95% CI: 1.02–1.65), received nutrition education (OR = 1.53, 95% CI: 1.25–1.86), had high academic performance (OR = 1.85, 95% CI: 1.34–2.57), and had a monthly household income of >¥12,000 (OR = 1.61, 95% CI: 1.14–2.26) were more likely to report a higher level of total NL than did other students. Regarding the six dimensions of NL, the results indicated that female students (OR = 1.35, 95% CI: 1.11–1.66) and medical students (OR = 1.56, 95% CI: 1.27–1.92) were more likely to report a higher level of nutrition knowledge than did other students. Older students (OR = 1.33, 95% CI: 1.05–1.68) were more likely to report a higher level of nutrition understanding than did other students. Students with high academic performance (OR = 1.52, 95% CI: 1.10–2.11) and a monthly household income of >¥12,000 (OR = 1.41, 95% CI: 1.01–1.97) were more likely to report a higher level of obtaining skills than did other students. In addition, students who were older (OR = 1.28, 95% CI: 1.01–1.62) and were from urban areas (OR = 1.27, 95% CI: 1.01–1.61) were more likely to report a higher level of applying skills than did other students. Students who were the only child in the family (OR = 1.29, 95% CI: 1.03–1.60) were more likely to report a higher level of interactive skills than did other students. Finally, female students (OR = 0.80, 95% CI: 0.66–0.98) were less likely to report a high level of critical skills than did male students.

**Table 2:** Multivariate analysis for variables associated with total nutrition literacy and its six dimensions.

**Characteristics**	**Nutrition literacy^a^**	**Knowledge^a^**	**Understanding^a^**	**Obtaining skills^a^**	**Applying skills^a^**	**Interactive skills^a^**	**Critical skills^a^**
**Age group (ref**. = **16–21)**							
22–27	1.14 (0.90, 1.44)	0.93 (0.74, 1.17)	1.33 (1.05, 1.68)^*^	1.21 (0.96, 1.53)	1.28 (1.01, 1.62)^*^	1.20 (0.95, 1.52)	1.14 (0.90, 1.44)
**Sex (ref**. = **Male)**							
Female	1.15 (0.94, 1.41)	1.35 (1.11, 1.66)^**^	1.09 (0.88, 1.33)	1.08 (0.88, 1.32)	1.13 (0.93, 1.39)	1.07 (0.88, 1.31)	0.80 (0.66, 0.98)^*^
**Major (ref**.=**Non-medical)**							
Medical	1.11 (0.90, 1.36)	1.56 (1.27, 1.92)^***^	1.72 (1.40, 2.13)^***^	1.16 (0.95, 1.43)	0.81 (0.66, 1.00)^*^	1.11 (0.90, 1.36)	1.02 (0.83, 1.26)
**Grade (ref**. =**Junior )**							
Senior	1.00 (0.80, 1.26)	0.96 (0.77, 1.20)	1.07 (0.85, 1.34)	1.00 (0.80, 1.25)	0.86 (0.68, 1.07)	0.90 (0.72, 1.12)	1.15 (0.92, 1.44)
**Place of origin (ref**. = **Rural)**							
Urban	1.36 (1.08, 1.72)^*^	1.32 (1.05, 1.67)^*^	1.24 (0.98, 1.57)	1.18 (0.94, 1.49)	1.27 (1.01, 1.61)^*^	1.22 (0.96, 1.53)	1.28 (1.01, 1.61)^*^
**Only-child status (ref**. = **No)**							
Yes	1.16 (0.93, 1.45)	0.98 (0.79, 1.23)	1.06 (0.85, 1.32)	1.18 (0.95, 1.47)	1.20 (0.96, 1.50)	1.29 (1.03, 1.60)^*^	1.15 (0.92, 1.43)
**Living arrangement (ref**. = **Living with a single parent, grandparents, or other)**							
Living with both parents	1.30 (1.02, 1.65)^*^	1.35 (1.06, 1.71)^*^	1.03 (0.80, 1.31)	1.06 (0.83, 1.35)	1.33 (1.04, 1.69)^*^	1.30 (1.02, 1.65)^*^	1.01 (0.79, 1.28)
**Father's education level (ref**. = **Elementary school or below)**							
Junior high school	1.11 (0.86, 1.44)	0.79 (0.62, 1.02)	1.11 (0.86, 1.44)	1.21 (0.94, 1.57)	1.06 (0.82, 1.36)	1.18 (0.92, 1.52)	0.98 (0.76, 1.27)
High school or technical school	1.09 (0.79, 1.50)	0.72 (0.52, 0.98)^*^	1.14 (0.82, 1.57)	1.17 (0.85, 1.61)	1.17 (0.85, 1.60)	1.14 (0.83, 1.56)	1.20 (0.88, 1.65)
College or university and above	1.01 (0.68, 1.49)	0.66 (0.44, 0.98)^*^	1.40 (0.94, 2.09)	1.19 (0.80, 1.76)	1.08 (0.73, 1.60)	1.06 (0.72, 1.57)	1.07 (0.72, 1.58)
**Mother's education level (ref**. = **Elementary school or below)**							
Junior high school	1.03 (0.83, 1.27)	1.00 (0.81, 1.24)	1.20 (0.96, 1.49)	1.09 (0.89, 1.35)	1.01 (0.82, 1.25)	1.04 (0.84, 1.28)	1.07 (0.86, 1.32)
High school or technical school	1.24 (0.91, 1.70)	1.29 (0.94, 1.77)	1.13 (0.82, 1.56)	1.01 (0.74, 1.39)	1.11 (0.81, 1.53)	1.17 (0.86, 1.60)	1.04 (0.76, 1.42)
College or university and above	1.24 (0.81, 1.91)	0.87 (0.56, 1.33)	1.16 (0.75, 1.79)	1.24 (0.81, 1.90)	1.22 (0.79, 1.87)	1.18 (0.77, 1.81)	1.41 (0.92, 2.17)
**Acquisition of nutrition education (ref**. = **No)**							
Yes	1.53 (1.25, 1.86)^***^	1.25 (1.03, 1.52)^*^	1.36 (1.11, 1.66)^*^	1.11 (0.91, 1.35)	1.42 (1.17, 1.74)^***^	1.33 (1.09, 1.62)^**^	1.50 (1.23, 1.82)^***^
**Academic performance (ref**. = **Poor)**							
Average	1.21 (0.88, 1.66)	0.96 (0.70, 1.32)	1.15 (0.83, 1.60)	1.08 (0.78, 1.48)	1.38 (1.00, 1.91)^*^	1.28 (0.93, 1.76)	1.07 (0.78, 1.48)
Good	1.85 (1.34, 2.57)^***^	1.16 (0.84, 1.60)	1.56 (1.12, 2.17)^**^	1.52 (1.10, 2.11)^*^	1.97 (1.42, 2.73)^***^	1.77 (1.28, 2.45)^**^	1.43 (1.03, 1.97)^*^
**Household income per month (RMB) (ref**.=** <6,000)**							
6,000-−12,000	1.08 (0.89, 1.31)	1.01 (0.83, 1.23)	1.13 (0.93, 1.38)	0.97 (0.80, 1.18)	1.05 (0.87, 1.28)	1.06 (0.88, 1.29)	1.04 (0.85, 1.26)
>12,000	1.61 (1.14, 2.26)^**^	1.27 (0.91, 1.78)	1.67 (1.18, 2.35)^**^	1.41 (1.01, 1.97)^*^	1.71 (1.22, 2.40)^**^	1.20 (0.86, 1.68)	1.20 (0.86, 1.68)
**Expenditure per month (RMB) (ref**.=** <1,000)**							
1,000-−1,500	1.04 (0.79, 1.37)	1.21 (0.92, 1.59)	1.08 (0.82, 1.43)	0.89 (0.68, 1.18)	0.82 (0.62, 1.08)	1.01 (0.77, 1.33)	1.08 (0.82, 1.42)
>1,500	0.98 (0.70, 1.37)	1.26 (0.90, 1.75)	1.25 (0.89, 1.76)	1.05 (0.76, 1.46)	0.68 (0.48, 0.94)^*^	0.97 (0.70, 1.35)	1.11 (0.79, 1.54)

OR represents the odds ratio, 95% CI represents 95% confidence intervals, and Ref. represents reference.

* *P* < 0.05,

** *P* < 0.01,

*** *P* < 0.001. Multiple logistic regression analysis was applied to estimate the OR and 95% CI for nutrition literacy.

a The final model is adjusted for age, sex, major, grade, place of origin, only-child status, living arrangement, father's education level, mother's education level, acquisition of nutrition education, academic performance, household income per month, expenditure per month.

The study also examined the interaction effects of multiple factors on total NL and its six dimensions (Table 3). The results revealed that the ORs for total NL (OR = 1.88, 95% CI: 1.26–2.81), nutrition understanding (OR = 1.73, 95% CI: 1.15–2.59), and critical skills (OR = 1.78, 95% CI: 1.19–2.66) were higher for medical students who were women than for other students. Furthermore, the ORs for total NL (OR = 1.60, 95% CI: 1.06–2.41), nutrition knowledge (OR = 1.51, 95% CI: 1.00–2.26), nutrition understanding (OR = 1.85, 95% CI: 1.22–2.80), obtaining skills (OR = 1.76, 95% CI: 1.16–2.65), and critical skills (OR = 1.59, 95% CI: 1.05–2.39) were higher for medical students who had received nutrition education than for other students. The ORs for total NL (OR = 2.42, 95% CI: 1.21–4.84), nutrition knowledge (OR = 2.69, 95% CI: 1.33–5.44), nutrition understanding (OR = 2.59, 95% CI: 1.28–5.25), interactive skills (OR = 2.06, 95% CI: 1.04–4.08), and critical skills (OR = 2.15, 95% CI: 1.09–4.28) were higher for students who were the only child in the family and had a monthly expenditure of >¥1,500 than for other students.

**Table 3:** The impact of multiple factor interactions on total nutrition literacy and its six dimensions.

**Characteristics**	**Nutrition literacy^a^**	**Knowledge^a^**	**Understanding^a^**	**Obtaining skills^a^**	**Applying skills^a^**	**Interactive skills^a^**	**Critical skills^a^**
**Major** × **Sex**							
Medical × Female	1.88 1.26, 2.81)^**^	0.75 (0.50, 1.12)	1.73 (1.15, 2.59)^**^	1.35 (0.90, 2.02)	1.15 (0.77, 1.72)	1.46 (0.98, 2.17)	1.78 (1.19, 2.66)^**^
**Major** × **Acquisition of nutrition education**							
Medical × Yes	1.60 (1.06, 2.41)^*^	1.51 (1.00, 2.26)^*^	1.85 (1.22, 2.80)^**^	1.76 (1.16, 2.65)^**^	1.09 (0.72, 1.64)	1.29 (0.86, 1.93)	1.59 (1.05, 2.39)^*^
**Only-child status** × **Expenditure per month (RMB)**							
Yes × 1,000-−1,500	2.21 (1.16, 4.21)^*^	2.38 (1.23, 4.60)^*^	2.01 (1.04, 3.89)^*^	1.17 (0.62, 2.21)	1.14 (0.60, 2.15)	1.48 (0.79, 2.79)	1.60 (0.85, 3.03)
Yes × >1,500	2.42 (1.21, 4.84)^*^	2.69 (1.33, 5.44)^**^	2.59 (1.28, 5.25)^**^	1.79 (0.90, 3.53)	1.04 (0.52, 2.06)	2.06 (1.04, 4.08)^*^	2.15 (1.09, 4.28)^*^

OR represents the odds ratio, 95% CI represents 95% confidence intervals, and Ref. represents reference.

^*^P < 0.05 and ^**^P < 0.01. Multiple logistic regression analysis was applied to estimate the OR and 95% CI for nutrition literacy.

a The final model is adjusted for age, sex, major, grade, place of origin, only-child status, living arrangement, father's education level, mother's education level, acquisition of nutrition education, academic performance, household income per month, expenditure per month.

## 4. Discussion

This study investigated factors associated with NL and its six dimensions among Chinese university students. The findings indicate that place of origin, living arrangement, acquisition of nutrition education, academic performance, and household income per month were independently associated with NL. This finding provides empirical evidence for designing nutrition literacy intervention strategies for practitioners, when implementing nutrition education in the future.

The application of food- and nutrition-related information acquired through a variety of media channels may be challenging for university students because of circumstances unique to university environments (19). In addition, excessive and ambiguous nutrition information may lead to confusion among individuals with low levels of nutrition knowledge (23). Therefore, the ability to exchange food- and nutrition-related information with family, peers, and experts or to extract information from different media channels, in addition to the quantity of such information, is crucial (13). NL might be an important factor in determining healthy-eating behaviors during university (25). Higher nutrition literacy, which is the immediate goal of nutrition education, would subsequently lead to higher diet quality (30). Therefore, advancing nutrition literacy in the school setting is important to promote healthy eating and support long-term academic outcomes to reduce the burden of food-related diseases across the lifespan (31). The present study demonstrated that older students were more likely to report higher levels of nutrition understanding and applying skills than did other students. A higher degree of cognitive abilities positively influences NL. Similarly, other factors such as practice, communication, media, and cognitive reserve may help increase NL in older students (32). The present study also revealed that female students were more likely to report a higher level of nutrition knowledge than did male students. Attitudes toward reading and learning generally differed by sex (33). Female students typically paid more attention to their dietary intake and were more likely to receive nutrition education than did male students (34). However, our findings reveal that female students had lower levels of critical NL than did male students. Women reported difficulty in distinguishing between scientific and non-scientific information on nutrition. Moreover, women were influenced by dietary advice presented in the media and considered alternative medical advice to be credible (16).

Nutrition and dietary information sources are associated with adequate NL (24). Students who received nutrition information at university, had taken nutrition-related courses, or had a strong demand for nutrition information exhibited superior NL (25). This result also strengthens the need for nutrition education on college campuses (25). The present study found that the ORs for total NL, nutrition knowledge, nutrition understanding, obtaining skills, and critical skills were higher for medical students who received nutrition education than for other students. The explanation for this finding is that in health science–related courses and professions—primarily nutrition and dietetics—topics such as nutrition, healthy eating, and correct eating habits are frequently addressed, as well as people who are accompanied by these professionals (35). Of course, It is also associated with the number of nutrition courses and nutrition education contents. As NL is literacy focusing on nutrition-related information, medical students with more exposure to medical and health information in faculty courses perform better compared to other non-medical courses (36). However, with the exception of students pursuing medicine-related majors, university students are unlikely to have access to nutrition education (21). NL encompasses a set of knowledge and competencies that an individual develops over the course of their life, and it can be regarded as an outcome of nutrition health education (37). Medical students who had received and applied more medical knowledge had more favorable objective conditions for acquiring nutrition knowledge and had a more comprehensive grasp of nutrition (38). Moreover, our findings suggest that students with high academic performance were more likely to report a higher level of total NL, nutrition understanding, obtaining skills, applying skills, interactive skills, and critical skills. Such students have a strong self-learning awareness and learning ability and a higher degree of absorption and understanding of nutrition information; this provides them with a foundation for screening and obtaining nutrition information in daily life and for developing healthy habits (38).

Social factors may affect NL and food- and nutrition-related decisions (39). This study revealed that students who were the only child in the family and had a high monthly expenditure reported higher NL, nutrition knowledge, nutrition understanding, interactive skills, and critical skills. In mainland China, only-child students are more likely to be from urban areas (40), and their families have higher monthly incomes (41). In addition, the parents of only-child students have a higher socioeconomic status (42). Under these superior family conditions, only-child students can receive more resources and support from their families, which can benefit their physical and psychological health (41). Individual NL is developed through information exchange with experts, peers, parents, or caregivers, and it is influenced by the context (37). Students from families with a lower socioeconomic status face barriers to developing a healthy diet; this is possibly because such families have fewer financial resources for the purchase of healthier foods or lack knowledge regarding nutritional recommendations for healthy eating (43). Students' ability to obtain, interpret, and apply information about nutrition affected their healthy eating behaviors (12). Our findings suggest that students from urban areas have a higher level of NL than those from rural areas; this is probably because rural students are affected by an underdeveloped economy, poor basic life outcomes, limited access to nutrition information, and low awareness of good eating habits. This demonstrates the importance of narrowing the gap between urban and rural nutrition services (34). Our results reveal no association between parental education level and the total NL level of university students. Family members of senior high school students usually focus more on students' study, resulting in insufficient nutrition education at home (34). Moreover, students who become independent in managing their diets are less affected by their parents from the time they enter university (25). This study observed that students who were living with both parents reported a higher level of NL. The eating behavior of individuals were indeed affected by their NL levels (23), and a higher NL level was associated with healthier eating practices and lifestyle behaviors (44). Two-parent families may have sufficient family functioning, which could lead to a higher family health status. By contrast, single-parent families may have fewer resources such as time, money, and social networks; this may lead to poor health outcomes (45).

This study has some limitations. First, it applied a cross-sectional design, which prevented the interpretation of the direction of the associations. Moreover, the results cannot be generalized to all students. Finally, this study used self-reported data, and discrepancies may have existed between the participants' subjective perceptions and actual practice, which may have led to errors in the interpretation of the results. Despite these limitations, this study adopted a rigorous research design, including valid and reliable measures and strict statistical procedures, to reduce possible research biases. Future research efforts need to investigate the association between nutrition literacy and nutrition-related diseases, and how to take targeted intervention to improve nutrition literacy.

## 5. Conclusions

This study revealed the NL of university students differed in terms of place of origin, living arrangement, nutrition education, academic performance, and household income. These findings imply that targeted intervention should consider the disparities of family resources and universities should have all students take a basic nutrition course to improve their NL.

## Data availability statement

The raw data supporting the conclusions of this article will be made available by the authors, without undue reservation.

## Ethics statement

The students were notified that participation was voluntary, and signed informed consent was obtained. This study was approved by the Ethics Committee of Bengbu Medical College.

## Author contributions

MZ and TG: conceptualization. HL, TG, and YD: methodology. HL, GM, and MZ: investigation and data management. TG: writing—original draft preparation. HL, YD, TG, and QQ: writing—review and editing. MZ, GM, and SH: supervision. HL, QQ, and SH: project administration. HL and MZ: funding acquisition. All authors contributed to the article and approved the submitted version.
