# Supplementary material for: Nutrition literacy differs based on demographics among University students in Bengbu, China
Source: Front Public Health. 2023 Mar 3;11:1113211. doi: 10.3389/fpubh.2023.1113211 (PMC10020653; doi:10.3389/fpubh.2023.1113211)
Supplement: Supplementary file 1 [file Table_1.DOCX]

Supplementary Material

# Supplementary Table

**Supplementary Table 1.** Total nutrition literacy and its six dimensions were dichotomised into low and high levels on the basis of their corresponding median scores

| Variables | Nutrition literacy level | |
| --- | --- | --- |
|  | low | high |
| Nutrition literacy | ≤150 | >150 |
| Knowledge | ≤29 | >29 |
| Understanding | ≤19 | >19 |
| Obtaining skills | ≤17 | >17 |
| Applying skills | ≤35 | >35 |
| Interactive skills | ≤29 | >29 |
| Critical skills | ≤19 | >19 |
